# Proceedings of the Annual Meeting of the Dental Association of Maryland and District of Columbia, September 7th and 8th, 1881

**Published:** 1881-12

**Authors:** 


AMERICAN JOURNAL

OF

DENTAL SCIENCE.

Vol. XV. THIRD SERIES—DECEMBER, 1881. No. 8.

### ARTICLE I.

*Proceedings of the Annual Meeting of the Dental Association of Maryland and District of Columbia, Held in Baltimore, September 7 and 8, 1881.*

FIRST DAY,
*Morning Session.*

The meeting was called to order by the President, Dr. H. B. Noble, at eleven o'clock.

Rev. Dr. Thomas Guard offered prayer, after which the meeting adjourned to attend the funeral of Dr. E. Lloyd Howard, late Professor of Chemistry in the Baltimore College of Dental Surgery.

*Afternoon Session.*

The President in the chair.

The following address of welcome was delivered by Dr. J. Emory Scott, of Baltimore—

*Mr. President and Gentlemen:*

Having been called upon at very short notice, to deliver the salutatory on this occasion, I find it necessary to begin my address with an apology, and I indulge the hope that the sincerity and cordiality of my intentions may atone for any and every deficiency in my execution.

While I ask your forbearance I give you in return one little drop of consolation—my effort shall be short if not correspondingly sweet.

Let me then, in the name of the dentists of Baltimore, and in the interests of our citizens generally, bid you a hearty welcome.

As members of this Society, we are indebted to each other for much help in ascending the "hills of difficulty" in our profession, and as dentists we have richly earned the gratitude and respect of the different communities in which we have exercised our healing art.

For, although the impression largely obtains among the laity, that dentistry is principally a "pulling" and "replacing" art, we have the every day satisfaction of knowing that our mission is to speak hope to the desolate, to comfort those who mourn, to give joy to the sorrowing, and to publish the glad tidings to a suffering world that dentistry is rapidly taking its rightful place in the minds as well as in the mouths of the people, and that the cultivated, at least, comprehend the difference between a skillful, educated dentist and the numerous "tooth carpenters" who from time to time, when refused admittance at the gates of our profession, impudently scale the walls to obtain entrance, and judging by their work "steal the livery of Heaven to serve the devil in."

However, we may safely leave these imposters to the public and to the certain Nemesis of time—

"The mills of the gods grind slowly,
But they grind exceeding small—
Though with patience they stand waiting,
With exactness grind they all."

As is our custom, we have assembled for an interchange of ideas and facts—to absorb and assimilate, and put in practice all that is good and useful in the experience of others, and to give freely any new thought or discovery of our own.

We have no desire to be fenced about with our own exclusiveness, and enriched only with the fertilizing influence of our individual theories—to take only such observations as our own limited views afford. We want a different treatment altogether; we want thorough and varied cultivation; we want "bone phosphates" dug out of other men's fields; we want our confined areas extended until they meet the broad acres of those who have labored long and faithfully in the dental vineyard, so that those who have come in at the eleventh hour, may share the experience and profit of those who have borne the heat and burden of the day.

To realize some of the benefits of our meetings we have only to examine the teeth of those people who do not know that there are *dentists*—and dentists.

They have, for the want of this knowledge, patronized men who can extract a tooth by main strength, who can put a plastic filling in a half prepared cavity—who are poor imitators of other poor dentists, unfortunate enough to have been the fathers or preceptors of these latter day humbugs.

They are not "old fogies"—who are much to be respected, when their ideas are *good* as well as *old*—but they are ignorant pretenders. To these, as well as to some better people the dental engine, the mallet, the rubber dam, cohesive foil, and the thousand and one inventions of late years remain mysterious and unknown. So numerous indeed have been inventions and improvements in dentistry that one must be constantly on the alert to keep abreast. Every new journal, every meeting brings some implement to perfect the work of the operative or mechanical dentist, and their merits and benefits are not confined to dentistry alone. We have given to surgery the god-like gift anæsthesia, thereby "steeping the surgeon's knife in the waters of oblivion"—the inter-dental splint, the most favored specialty of surgery, entirely doing away with the old treatment, requiring ninety feet of bandages and the knocking out of the front teeth for the purpose of administering food—the dental engine modified to be used as a surgical engine in cranial operations, and in many ways is surgery under lasting obligations to the profession of dentistry—which still declines however to be regarded as a specialty of medicine; prefering to be considered rather as the late Dr. Fuller described it, *one* of the *exact sciences*.

Let us then continue in the path set before us, not only with alacrity and delight but with credit to our own profession and benefit to other professions and to the world at large. Let us not forget that while we labor for those we love we labor also for the healing of the nations.

Once more I bid you a cordial welcome.

Dr. Noble, President, then delivered the following address:

*Members of the Maryland and District of Columbia Dental Association:*

Gentlemen.—It is now nearly seven years since the organization of this Society. In looking around I miss many of the faces then present, and not as many new ones as we had reason to believe would be glad to come in and work with us. We all then had high hopes of what we would, as a Society, be able to accomplish for the good of our profession; some of the work in the way of gathering statistics by or through the general government, we found to be of a more national scope and therefore more properly belonging to a national organization; and we confidently anticipate that the National Dental Association of the United States of America, organized at New York last year, and which is to meet every four years in Washington, D. C., will take hold of this very important subject with vigor, as the officers of the United States government have promised their active co-operation and assistance in obtaining these statistics and taking charge of all collections, so that at no distant day we may with confidence look for a valuable and interesting library and museum at the National Capitol.

We would advise each individual member of this Society to give the new organization his attention and influence.

Our Society took the lead in organizing into sections, but their internal work and reports have not been up to what was expected; we think this is in a large measure owing to a want of bringing the members of the sections into contact with each other so there may be a better understanding of each other's labors and views. To overcome this there should be quarterly meetings of the sections, to discuss such questions as the chairmen or any members may suggest. By these meetings and discussions the members would become better acquainted with each other's views and opinions, and the chairman would be able at each annual meeting to report the views, opinions, inventions or appliances of his section, so that it would not as now, be only the individual report of the chairman. The effort which has been made to get a written report from each member of a section to its chairman has signally failed, but we think they would attend and give their verbal reports to quarterly meetings. I would urgently recommend its trial, and hope some definite action to this end will be taken at this meeting. We believe such meetings would so interest members that we would have larger numbers at our annual gatherings, for it must be apparent to all, that we have failed to interest a large number who ought to be here with us, and who would be, if they had assurance of interesting and profitable reports. There are always numbers who for various reasons are standing in their own light ready to receive but not to give.

One of our members told me he was not coming to this meeting because a paper he had prepared with great care had been passed without discussion. Why was this? Mostly because of its labor and research it covered ground and touched points but little thought of by the members, consequently, they were not prepared to intelligently discuss them; now a quarterly meeting of his section as recommended, would have prepared the members to speak on the subject, cheered the writer, and shown an appreciation of his labors that would have inspired him to give us more work in the same direction.

Many think that clinics should be a more prominent feature of State meetings. They would without doubt be a grea attraction to many, and when meeting in such a building as this, they ought to be on a more extended scale, and go beyond the filling of a few simple cavities, but should cover and illustrate any new operations, or modes of practice, in both the surgical and operative departments, so that every dentist would feel that he had seen, as well as heard, something to his advantage. Many operations can only be thoroughly comprehended—in fact most operations are better understood by seeing them performed. Most local societies meet evenings so that it is not practical to have clinics. National societies may very properly look more to the scientific aspect of questions and have not the time for practical operations, so that the State societies would seem to be the especial place for the illustrations of theories or special modes of practice; particularly when surrounded by all the appliances of a Dental College such as we have here in the Baltimore College of Dental Surgery, probably the most perfect of its kind in the world. Have we not reason to expect some practical illustration of the manner in which it is educating the young men of the profession. Such operations and clinics would furnish abundant topics for interesting and profitable discussion, and in many cases, clear up doubts and lead to the better understanding of the various modes of practice, and help us to a better understanding and confidence in ourselves. I would call the attention of the Society to a fact that may not have been generally observed—that all good clinical operators are sought after by societies, not alone for their operations, but because working before their brethren gives them confidence in themselves and begets a kind of faith that makes them good talkers as well as workers. We think discussion and debate far more interesting than long prosy papers; the expecting and requiring of long papers and essays has without doubt, hurt many a dental society. Short papers upon some single point rather than the long essay would, I am sure, be for the interest and good of our Society. Let us take as an example our daily papers, with their short crisp editorials, and studying to all how short they can make an article in contrast to the old long ponderous editorial, so that we would come to these meetings with the same interest we read our morning papers.

It is a valuable lesson to learn to be concise in what we have to say and stop when it is said.

Most of the work and business of our Society seems to to fall upon the older men. It has been remarked that the old fellows get all the honors, and they ought to do the work. Will not this Society agree to look after its young men and give them a chance?

The cities of Baltimore and Washington have the opportunities for a larger experience than most other places. At the Capitol we have patients from every State and district in the country, and of seeing work from the dentists all over the world, and comparing all kinds and styles of work, and by comparison, see what kinds of work stand the best, this is becoming more and more understood and our advice and opinions sought; let us then as a society be very careful of our recorded opinions, that they are founded upon correct opinions and a careful judgment.

For the first time in several years we find ourselves clear of debt, and let us keep so, for these assessments have without doubt kept many a worthy young man from becoming a member. Let us examine all who make applications carefully, that no unclean hands may be found among us. We had hoped during the past year to have had some laws passed by Congress and the State of Maryland for the better protection of our profession and the public, against the imposition of empirics and ignorant men who have been driven from other portions of our country by proper laws regulating and governing the practice of dentistry. The reasons for the failure to secure such legislation will probably be given, and your aid and assistance asked by the section on education, so we may not be behind our sister States in this respect.

That dread summons from the eternal world has called one of our number to his final account, one whom we all loved and respected. His warm heart and brilliant mind made him a recognized power in our councils, and he was one of the first of our presiding officers. We shall sadly miss his cheering presence from our meetings, as he was one of the faithful few whose face was sure to be seen. I refer to Byron F. Coy, D. D. S., whose sudden death occurred last July.

Having committed myself to the doctrine of short addresses, I will now leave the subjects brought to your notice in your hands, asking that all mistakes as your presiding officer will be excused, as it is my wish only to serve the best interests of the society over which I feel it an honor to preside.

On motion of Dr. J. E. Scott, Dr. Coyle was se lected treasurer *pro tem.*

On motion of Dr. Hunt the reading of the proceedings was dispensed with.

Drs. W. G. Foster, Benj. Flannigain and B. Holly Smith, were unanimously elected members.

Dr. Winder said that at the meeting held last year in Washington, the committee appointed to aid in getting a bill through the Legislature for the better protection of dentists, was continued, and asked if it was the will of the Association that that committee be still continued.

Dr. Foster moved that the committee be continued, and that a member be appointed on the committee to fill the vacancy caused by the death of Dr. B. F. Coy. Adopted.

Dr. Winder, as Chairman of the Executive Committee, reported that the weather was so hot that even the public schools had adjourned, and said it had been suggested that this meeting adjourn until to-morrow morning. Adjourned.

SECOND DAY,
*Morning Session.*

Clinics by Drs. T. S. Waters and Benj. Flannigain, at 9 A. M.

The Association met at 11 A. M., the President in the chair.

Dr. A. Stewart Koser was elected a member of the Society.

Dr. H. M. Schooley, Chairman ex-officio of Publication Committee, made the following report, which was adopted :

The Publication Committee have to report that through the kindness of Professors Gorgas and Hodgkin, editors, and Messrs. Snowden & Cowman, publishers of the AMERICAN JOURNAL OF DENTAL SCIENCE, the proceedings of the meeting of the Society, held in Washington, in October, 1880, were satisfactorily printed in that journal.

H. M. SCHOOLEY,

*Ch'man Ex-officio.*

Dr. Winder reported the Association entirely out of debt.

Dr. M. F. Thompson, of Washington, was proposed for active membership and was unanimously elected.

Dr. Winder moved that a committee of three be appointed to draft suitable resolutions expressive of the feelings of the members of the Association in regard to the death of Dr. B. F. Coy. The President appointed on that committee Drs. Winder, Hunt and Foster, who presented the following obituary, which was ordered to be placed upon the minutes. :

OBITUARY.

This Association learns with deep sorrow of the loss of our beloved brother and fellow associate, Dr. Byron F. Coy, who has been removed from our midst by an untimely death.

He was one of the original members of this Association, and did much towards its formation and organization ; indeed, the better part of his life was unselfishly spent in trying to advance the best interests of the dental profession. Professionally he was a tower of strength, and his many manly qualities endeared him to us socially. We shall miss him sadly. We tender our sincere sympathy to his family in their great bereavement, and assure them of our undying interest in their future welfare and happiness.

Dr. Winder offered the following resolution :

*Whereas*, The National and Southern Dental Associations meet the ensuing year respectively in the Cities of Washington and Baltimore, and earnestly desiring to put forth our best energies to aid in making these meetings successful and satisfactory, it is thought advisable to dispense with the regular meeting of this Association for the next year ; therefore

*Be it Resolved*, That the Dental Association of the State of Maryland and District of Columbia elect officers to hold over for two years, or until our next meeting in Baltimore. The President however having the power of calling a meeting at any time in the interim should the interest of the Association demand it ; and that when we adjourn, we adjourn to meet in Baltimore, on the last Monday in October, 1883.

On motion of Dr. Winder a committee of three, consisting of Drs. Winder, Hunt and Donaldson, was appointed to revise the Constitution and By-laws of this Society.

Dr. Hunt moved that on account of the extreme warm weather, such papers as were to be read at this meeting, be held over until the meeting of the Southern Dental Association in Baltimore next year, and presented at that time. The motion was adopte.d

He then offered the following resolutions which were adopted :

*Resolved*, That this Association has viewed with deep interest the efforts which culminated in the formation last year, of a National Dental Association, and that that

Association, based as it is on the broadly national plan and principles set forth in its published constitution meets with the hearty approval and will receive the cordial support of this body.

Dr. Hunt also moved that the dues of the Secretary be remitted, and an appropriation of ten dollars be made as part pay for his services as stenographer. Carried.

Dr. Foster, Chairman of the Scholarship Committee read applications for the Scholarship in the Baltimore College of Dental Surgery, from two gentlemen, one from Maryland and one from the District of Columbia.

Dr. Hunt moved that for the guidance of the Scholarship committee, it shall be the sense of the Association that the Scholarship be conferred alternately upon candidates from Maryland and the District of Columbia.

Dr. Foster amended by making it so that if there be more than one candidate from the same place that a competitive examination be held. The motion was carried as amended.

The Scholarship was then on the report by the committee conferred on Mr. W. H. Deford, of District of Columbia.

Dr. Winder moved that the officers of the Association have the power to give credentials to any members wishing to visit meetings of any other Associations. Adopted.

On motion of Dr. Winder, the Secretary was given authority to have the seal of the Association changed, by substituting "Association" for "Society." Adopted.

Also that the present Chairmen of the Sections be continued. Adopted.

The Association then went into the election of officers with the following result:

*President*—Dr. T. S. Waters, of Baltimore.

*Vice President*—Dr. H. C. Thompson, of Washington.

*Rec. Secretary*—Dr. H. M. Schooley, of Washington.

*Rep.* " Dr. J. Emory Scott, of Baltimore.

*Treasurer*—Dr. W. G. Foster, of Baltimore.

*Executive Committee*—Drs. R. B. Winder, Jr., A. S. Koser, and Hall Lewis.

The retiring President before leaving the chair said he was happy to welcome the newly elected president and felt that he was turning over the Association into good hands; and thanked the members for their courtesy extended him during his term of office.

Dr. Waters.—The retiring President has rather flattered me in saying that he has no doubt that he has turned over the Association into hands that are fully competent to manage it. I am very much indebted to you for the honor conferred upon me. I will endeavor to do the best I can and ask your co-operation and assistance.

Dr. Thompson.—All I can say is, I am sorry I cannot express admiration of your choice as Vice President, but as such I promise to give the President such co-operation as he may desire.

A vote of thanks was tendered the retiring President, the Faculty of the Baltimore College of Dental Surgery, the press, Rev. Thomas Guard, and the editors and publishers of the AMERICAN JOURNAL OF DENTAL SCIENCE.

The President appointed the following committees:

*Publication*—Drs. R. F. Hunt and J. B. Hodgkin with H. M. Schooley, Chairman *Ex-officio*.

*Scholarship*—Drs. W. W. Evans, C. E. Duck, and J. E. Scott.

*Newspaper Articles*—Drs. R. B. Donaldson, R. F. Hunt, J. C. Smithe, R. B. Winder, E. P. Keech and M. W. Foster.

The meeting then adjourned.

